# HBS-Tools for Hairpin Bisulfite Sequencing Data Processing and Analysis

**DOI:** 10.1155/2015/760423

**Published:** 2015-12-20

**Authors:** Ming-an Sun, Karthik Raja Velmurugan, David Keimig, Hehuang Xie

**Affiliations:** ^1^Epigenomics and Computational Biology Lab, Virginia Bioinformatics Institute, Virginia Tech, Blacksburg, VA 24061, USA; ^2^Interdisciplinary Ph.D. Program in Genomics, Bioinformatics, and Computational Biology, Virginia Tech, Blacksburg, VA 24061, USA; ^3^Department of Biological Sciences, Virginia Tech, Blacksburg, VA 24061, USA

## Abstract

The emerging genome-wide hairpin bisulfite sequencing (hairpin-BS-Seq) technique enables the determination of the methylation pattern for DNA double strands simultaneously. Compared with traditional bisulfite sequencing (BS-Seq) techniques, hairpin-BS-Seq can determine methylation fidelity and increase mapping efficiency. However, no computational tool has been designed for the analysis of hairpin-BS-Seq data yet. Here we present HBS-tools, a set of command line based tools for the preprocessing, mapping, methylation calling, and summarizing of genome-wide hairpin-BS-Seq data. It accepts paired-end hairpin-BS-Seq reads to recover the original (pre-bisulfite-converted) sequences using global alignment and then calls the methylation statuses for cytosines on both DNA strands after mapping the original sequences to the reference genome. After applying to hairpin-BS-Seq datasets, we found that HBS-tools have a reduced mapping time and improved mapping efficiency compared with state-of-the-art mapping tools. The HBS-tools source scripts, along with user guide and testing data, are freely available for download.

## 1. Introduction

During cell division, DNA methylation patterns are faithfully copied from the parental to daughter strands by DNA methyltransferase 1 [1, 2]. Although most cytosines at CpG dyads were found to be either symmetrically methylated or completely unmethylated, asymmetric DNA methylation at certain genomic loci has been found to be associated with stochastic methylation changes in normal tissues and contribute to the epigenetic heterogeneity and eventually to phenotypic diversity [3]. In addition, increased asymmetric methylation was frequently observed in tumors with unstable epigenomes [4].

To determine the symmetry of CpG methylation, Laird and colleagues developed a hairpin bisulfite PCR technique to generate methylation data for both complementary strands simultaneously [5]. Recently, we implemented a genome-wide hairpin-BS-Seq technique to enable the assessment of global methylation fidelity [6]. In brief, genomic DNA was extracted and then sonicated into short fragments and ligated to the biotinylated hairpin and Illumina sequencing adaptors simultaneously. Following the streptavidin-capture and bisulfite PCR, the fragments linked to both the hairpin adaptor and Illumina sequencing adaptor were amplified for high-throughput paired-end sequencing. Compared to traditional BS-Seq strategies, hairpin-BS-Seq provides several advantages apart from assessing methylation inheritance fidelity: (1) unlike traditional BS-Seq techniques which result in reduced sequence complexity, the possibility of recovering the original (pre-bisulfite-converted) sequence from hairpin-BS-Seq data to improve mapping efficiency; (2) the ability to accurately determine the SNPs including C-to-T conversion; (3) the estimation of PCR and/or sequencing errors by examining the mismatches (excluding C-to-T and G-to-A mismatches which could result from bisulfite conversion) between read1 and read2.

In the past years, great efforts have been made to develop excellent algorithms and tools for the processing and analyzing of traditional BS-Seq data [7–10] but none for hairpin-BS-Seq data. In this study, we designed and implemented HBS-tools and compared them against other state-of-the-art mapping tools. Our result indicated that HBS-tools have a reduced mapping time and improved mapping efficiency.

## 2. Software Description

HBS-tools include a set of scripts (implemented in PERL and C) for the processing and analysis of hairpin-BS-Seq data (Table 1). The functions of core modules are described as below.

### 2.1. hbs_process

The hbs_process is designed for the processing of hairpin-BS-Seq data. It takes raw fastQ files from hairpin-BS-Seq as input and integrates functions including (1) trimming bad quality residues from the input sequence; (2) filtering hairpin adaptors; (3) filtering sequencing adaptors; (4) discarding read pairs with any read shorter than the given threshold after (1)–(3) steps.

### 2.2. hbs_mapper

**Figure 1:**
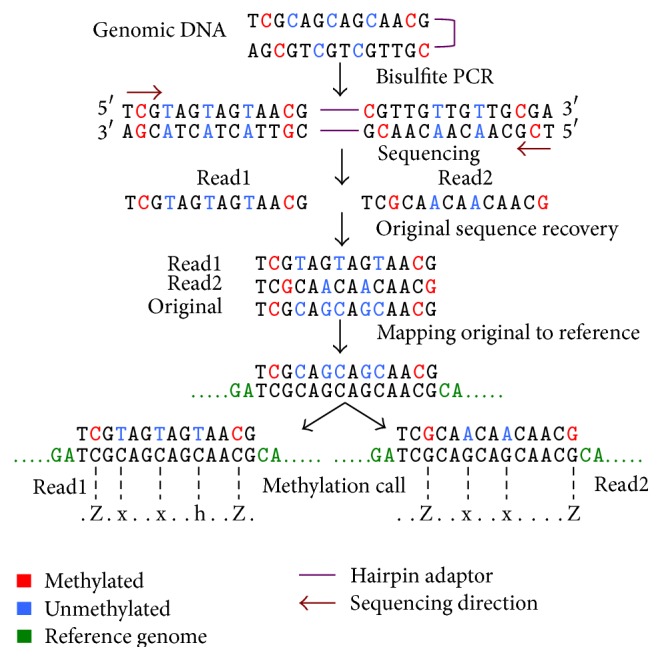
Genome-wide hairpin bisulfite sequence generation and processing. The flow chart begins with double stranded genomic DNA ligated to hairpin adapter. Hairpin bisulfite PCR products are sequenced from both ends. HBS-tools accept these pair-end hairpin-BS-Seq reads to recover the original (pre-bisulfite converted) sequences. The recovered sequences are aligned with the reference genome. Methylation calls are obtained based on the sequence alignment between raw sequence reads and corresponding genome sequences.

The hbs_mapper is the program for mapping hairpin-BS-Seq reads to the reference genome and obtaining methylation calls subsequently. Unlike previously published tools which usually map bisulfite-converted reads to reference genomes after C-to-T and G-to-A conversion of both reads and reference genomes, hbs_mapper fully takes advantage of the merits of hairpin-BS-Seq reads and utilized a special recover-then-mapping strategy for read mapping. In brief, it first recovers the original sequences after global alignment of read1 and read2 with the Needleman-Wunsch algorithm using a modified scoring matrix which tolerates the inconsistence between read1 and read2 probably due to bisulfite conversion (e.g., C-to-T in read1 and G-to-A in read2). After trimming the overhangs for the alignment at the two ends which may be due to different length of read1 and read2 and/or sequencing errors, the recovered sequences are mapped to the reference using Bowtie1 or Bowtie2 [11, 12] (Figure 1). Such a mapping procedure overcomes the reduced sequence complexity which is evident for traditional BS-Seq and thus improves mapping efficiency.

After global alignment of read1 and read2, the original sequence is recovered by following four simple rules: (a) a T in read1 and a C in read2 represent a C-to-T conversion during bisulfite treatment and hence the original sequence must have had a C; (b) a G in read1 and an A in read2 represent a G-to-A conversion and hence the original sequence must have had a G; (c) when read1 and read2 have the same nucleotide it represents no modification and hence stays the same in the recovered original sequence; (d) when read1 and read2 have different nucleotides that are not due to C-to-T or G-to-A conversion, the one with the better quality score will be kept. The recovered original sequence is then mapped to the reference genome using Bowtie1 or Bowtie2 [11, 12]. Having tracked the reference genome fragment that corresponds to the original sequence, the raw read1 and read2 are compared to the reference genome fragment to call the methylation statuses for covered cytosines.

The methylation calls and the alignment information are generated in standard SAM format [13]. The output contains information such as read ID, chromosome, genomic position, and methylation calls. The methylation call string is designed in a fashion so as to represent the methylation statuses of cytosines in three possible contexts. The small and capital letters of “z,” “x,” and “h” are used to represent the unmethylated and methylated events at CpG, CHG, and CHH sites, respectively. The mapping output can be used for postprocessing to extract methylation call information for individual cytosines.

### 2.3. hbs_methylation_extractor

The hbs_methylation_extractor takes the SAM file generated by hbs_mapper as input, parses the methylation call strings, and extracts the methylation statuses for the cytosines covered by hairpin-BS-Seq reads. It provides the options to output methylation information for CpG and non-CpG contexts either separately or together.

In the hbs_mapper output, each line represents the mapping and methylation call for a sequence read. In the extractor output, each line contains the information for the methylation status of one cytosine covered by a sequence read. Apart from the read ID and methylation status of a cytosine, the extractor output also contains chromosome, genomic coordinate, and strand information.

### 2.4. hbs_cg_mlmf and hbs_ch_ml

In addition to the methylation pattern obtained from each read, we are also interested in the methylation patterns for CpG dyads along the genome. Thus two simple yet useful scripts, hbs_cg_mlmf and hbs_ch_ml, were designed to summarize the methylation patterns for CpG and non-CpG sites, respectively. hbs_cg_mlmf takes the CpG methylation callings generated from the hbs_methylation_extractor to calculate the methylation level, methylation fidelity, and other related information for each CpG site determined. Similarly, hbs_ch_ml takes the non_CpG methylation calling result as input to calculate the methylation level for each covered non_CpG site. The outputs of two scripts can be used for further comparison between different samples.

## 3. Software Performance

To test the performance of HBS-tools, we applied hbs_mapper to hairpin-BS-Seq data [6] retrieved from NCBI Sequence Read Archive (SRA) database with accession numbers SRR919303, SRR919304, SRR919305, and SRR919306. These hairpin-BS-Seq data were generated for self-renewal mouse embryonic stem cells (ESCs) using the Illumina HiSeq 2000 platform. All reads are of 101 bp in length. The bad quality bases, hairpin adaptor, and sequencing adaptor were trimmed using the hbs_process, and read pairs shorter than 50 bp after trimming were discarded. Finally, we obtained 31.4 M, 31.8 M, 31.8 M, and 31.7 M read pairs for these four datasets, respectively. The reference genome (mm10) was downloaded from the UCSC genome browser [14].

The processed datasets were then used as the benchmark for the comparison between hbs_mapper and Bismark [9], which is the most widely used aligner and methylation caller for BS-Seq data. Notably, both hbs_mapper and Bismark used Bowtie as the engine for mapping. The analysis was conducted on a large-memory server of 12-core 2.90 GHz Intel Xeon CPU that runs SUSE Linux operating system. For unbiased comparison, the same parameters (-*n* 2 -*l* 50) for Bowtie were used for hbs_mapper and Bismark. Read1 and read2 were mapped separately using Bismark, because hairpin-BS-Seq data is different from traditional paired-end BS-Seq data and cannot be mapped using the paired-end mode by Bismark. The result indicated that hbs_mapper is more time-efficient and could achieve improved mapping efficiency for hairpin-BS-Seq data.

### 3.1. Running Time

We compared the running times for mapping hairpin-BS-Seq data. The result showed that hbs_mapper is more time-efficient compared with Bismark. In average, hbs_mapper uses around 35.8% less CPU hours compared with Bismark. This is because hbs_mapper maps the recovered original sequence to the reference genome using one thread, while Bismark needs to map the C-to-T and G-to-A converted reads to the reference genome using two (directional DNA library) or four (nondirectional library) threads.

### 3.2. Mapping Efficiency

**Figure 2:**
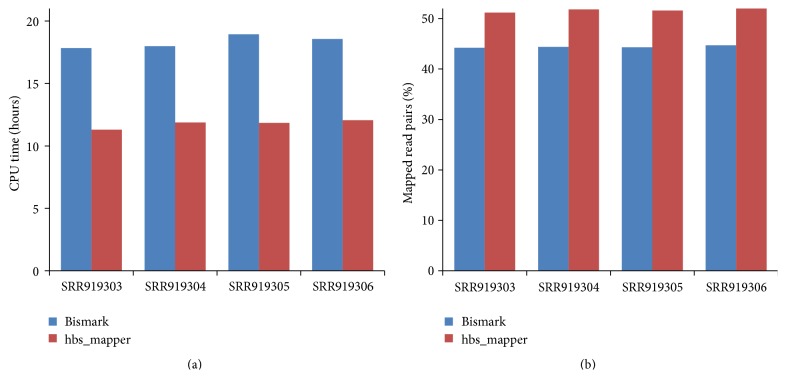
Comparison of the running times and mapping efficiencies between HBS-tools and Bismark. (a) CPU time used by Bismark and hbs_mapper for the mapping of public hairpin-BS-Seq datasets to reference. (b) The percentage of hairpin-BS-Seq read pairs mapped by Bismark and hbs_mapper, respectively.

We first compared the percentages of read pairs mapped by hbs_mapper and Bismark. For Bismark, read1 and read2 were first mapped separately to the reference genome, and then mapped read pairs were determined as those with both read1 and read2 mapped to the same chromosome and are less than 50 bp away. By doing it this way, around 44.4% of read pairs could be mapped by Bismark (Figure 2(a)). When using hbs_mapper, around 51.7% of read pairs could be mapped to reference (Figure 2(b)). We next asked if more reads (read1 or read2) could be mapped by combining hbs_mapper and Bismark. The result showed that while around 55.8% (between 55.7% and 56.0%) reads could be mapped by using only Bismark, 60.9% (between 60.8% and 61.1%) were mapped by combining the results of hbs_mapper and Bismark.

## 4. Discussion

Traditional BS-Seq cannot determine hemimethylation and also suffers from mapping errors due to reduced DNA complexity. In contrast, hairpin-BS-Seq allows the determination of methylation information from both DNA strands simultaneously. Therefore, hairpin-BS-Seq could not only be used to assess methylation fidelity, but also have the potential to improve mapping efficiency by recovering original sequences from the read pairs. Here we described HBS-tools, a set of programs specially designed for the analysis of genome-wide hairpin-BS-Seq data. When applied to real hairpin-BS-Seq data, the result indicated that HBS-tools are more time-efficient and have improved mapping efficiency compared with similar tools designed for traditional BS-Seq data.

## Figures and Tables

**Table 1:** Summary of the programs included in HBS-tools.

Module name	Function
hbs_process	Preprocessing of raw reads, including bad quality bases trimming, sequencing adaptor, and hairpin adaptor removal
hbs_mapper	Original sequence recovery, mapping, and SAM file output
hbs_methylation_extractor	Extract and output methylation pattern from the SAM file
hbs_cg_mlmf	Summarize the methylation level and fidelity for covered CpG sites
hbs_ch_ml	Summarize the methylation level for covered non_CpG sites
